# Management behaviors during the COVID-19 pandemic: The case of healthcare middle managers

**DOI:** 10.3389/fpsyg.2022.986980

**Published:** 2022-11-03

**Authors:** Marie-Christine Mackay, Marie-Hélène Gilbert, Pierre-Sébastien Fournier, Julie Dextras-Gauthier, Frédéric Boucher

**Affiliations:** ^1^Department of Psychology, Université de Sherbrooke, Sherbrooke, QC, Canada; ^2^Department of Management, Université Laval, Quebec, QC, Canada

**Keywords:** management behaviors, COVID-19, crisis management, healthcare sector, pandemic, middle managers

## Abstract

**Background:**

The spread of COVID-19 has disrupted the lifestyles of the world’s population. In the workplace, the pandemic has affected all sectors and has changed the way work is organized and carried out. The health sector has been severely impacted by the pandemic and has faced enormous challenges in maintaining healthcare services while providing care to those infected by the virus. At the heart of this battle, healthcare managers were key players in ensuring the orchestration of operations and the physical and mental availability of employees during the crisis. Although few studies have been conducted to identify organizational practices or leadership skills to be adopted in a crisis context, the concrete behaviors of managers have not been documented yet. Therefore, this study aims at filling this gap by studying middle managers’ behaviors facing COVID-19 crisis in the healthcare sector.

**Methods:**

Using a qualitative approach, eight focus groups were conducted online during the pandemic with 37 middle managers from the healthcare community of a Quebec health establishment (Canada) from April to June 2020. Thematic analyses were conducted, and a mixed-methods approach was used to analyse the data based on Viitala’s hierarchical model of management skills.

**Results:**

Based on the six managerial skills proposed in the model of Viitala, 21 specific management behaviors were identified as having been deployed by middle managers at the beginning of the pandemic. Considering that the health sector has been profoundly shaken by this health crisis, in addition to being an environment likely to experience other crises, managers need to develop practical skills in various crisis management situations. Thus, the results guide practitioners by highlighting the importance of team-oriented management behaviors (leadership, supervisory competencies), especially in a crisis context.

## Introduction

In December 2019, the first case of coronavirus disease (COVID-19) was reported in the city of Wuhan, China (World Health Organization, 2021). Since that day, this disease has spread around the world causing the death of hundreds of thousands of people, placing the world population in confinement and testing the limits of health systems around the world (Gopinath, 2020). Indeed, this pandemic has affected several sectors, in particular the health sector, which has found itself facing enormous challenges in countering the global spread of the coronavirus (Holge-Hazelton et al., 2021). Faced with the arrival of the pandemic in Quebec in 2020, the government implemented civil security structures to increase the level of preparation, particularly in the health sector, to deal with the health crisis (Ministry of Quebec Public Security, 2020). In response to the advent of this crisis in healthcare settings, managers had to restructure their organizational practices by, for example, abandoning less urgent medical procedures and other unit operations, establishing a safe environment, and offering infected patients the best care possible (Holge-Hazelton et al., 2021). On the one hand, although healthcare managers are used to frequent and continuous changes in their daily lives, this pandemic has forced them to go through out of the ordinary workloads for which their roles, skills and values have been put to the test (Rakowsky et al., 2020; Saltman, 2020). On the other hand, when a crisis occurs on the organizational level, despite the level of uncertainty present, it offers an opportunity for learning, capacity development or reconsideration of usual practices to overcome possible crises and important issues (Hatum et al., 2010; Williams et al., 2017). From this perspective, Galanti (2021) investigated risk management in the emergency context due to the COVID-19 pandemic. More specifically, the study examined the perception of workplace errors and the possibility to learn from errors to understand how organizations and leaders can best manage the learning climate and improve knowledge in a fast-changing world that requires employees to be adaptable, agile and flexible. Indeed, the preliminary results of Galanti’s study seemed to confirm the urgency and relevance for organizations to extend the advantages of Error Management Training in a crisis context.

According to Lockwood (2005), managers play key roles in a crisis, whether to establish a climate of trust, maintain effective communication or ensure productivity. Thus, their skills in a crisis context are essential to ensure the success of crisis management (Lockwood, 2005). Moreover, middle managers play a key role as a junction point for the transfer of information between senior management and employees in the field (Pappas et al., 2004). The fact that not only do they know the strategies of the organization, but also the social structure in place, allows them to stay in touch with ongoing operations while ensuring the well-being of employees, especially in times of change (McConville and Holden, 1999; Pappas et al., 2004).

So far, it is important to emphasize that authors have reported the characteristics, personality traits and qualities of leaders (Mumford et al., 2007; Panos et al., 2009; Bajaba et al., 2021), also types of leadership (Mustajab et al., 2020; Suprapti et al., 2020; Garretsen et al., 2022) or even cognitive resources needed in time of crisis (Maykrantz et al., 2021). For example, Garretsen et al. (2022) explored how large exogenous shocks like COVID-19 can impact leadership behavior and found that during the first lockdown of the pandemic, directive leadership increased significantly. In another study, Suprapti et al. (2020) concluded, during the COVID-19 pandemic, that transformational leadership and organizational climate had a positive and significant influence on work performance. Moreover, Bajaba et al. (2021) argued that managers with an adaptive personality exhibit a tendency to have a higher level of self-efficacy, resulting in increased motivation to lead in times of crisis. From another perspective, Maykrantz et al. (2021) explored the potential role of self-leadership and psychological capital as important cognitive resources to develop health-protective attitudes and behaviors, especially during current and future pandemics. In addition, other studies have focused on the notion of complexity that organizations increasingly face in their daily operations, particularly because of the pressure placed on them to innovate and develop continuously (Lewis, 1994; Liu et al., 2022). In this sense, Uhl-Bien (2021) takes COVID-19 as an example to illustrate the complexity that organizations must face. One of the key elements raised by the author is to put forward leadership behaviors, which allow individuals and organizations to better adapt to such situations. For example, why did some leaders in the same organization react well and respond well to COVID-19 while others did not? Thus, although some authors (Smits and Ezzat Ally, 2003; Mbolekwano, 2016) have been interested in the issues encountered by managers in a crisis context and others have been interested in the importance of their leadership and their personal characteristics in this type of situation (Lewis, 1994; Mumford et al., 2007; Bajaba et al., 2021; Liu et al., 2022), the fact remains that few studies have focused on documenting the behaviors actually expressed by managers in the context of a crisis. Recently, Caringal et al. (2021) examined the perspective of employees to identify the behaviors of effective leaders during the COVID-19 crisis. However, in their study, only the point of view of employees was solicited, the study was carried out in different sectors of activity likely to have been affected at different levels by COVID-19 in addition to being carried out specifically in the Philippines.

Thus, considering that (1) certain behaviors expressed by managers cannot be directly observed by their employees (e.g., managing priorities) and that the perspective of managers on their own behaviors is important to examine (Saint-Hilaire, 2012), (2) the current COVID-19 crisis has particularly impacted health network managers (Holge-Hazelton et al., 2021), (3) countries have been affected differently by this health crisis (Haldane et al., 2021) and (4) the specific behaviors of managers are particularly important to study if we want to offer proper guidance in the development of future managers (St-Hilaire, 2012; Gilbert et al., 2017), this study aims to give a voice to managers in the Quebec health network to document the specific behaviors expressed during the COVID-19 crisis.

### Conceptualization of management behaviors

To fully understand and conceptualize management behaviors within the study, three models will be presented: Boyatzis’s model (1982), Whetten and Cameron’s model (2005) and Viitala’s model (2005). First, it is important to highlight the lack of consensus in the literature on the concept of competence, which has often been reported in different ways (Boyatzis, 1982). Although the definitions of competencies tend towards the same concept, i.e., characteristics which, when present in an individual, are associated with better performance, the concept of competence remains a terminological blur in the literature (Benomar and Fortin, 2020). Since this study focuses on management competency models, which refer to the skills, practices, competencies, or abilities of an individual to be effective in his or her managerial role (McCauley and Van Velsor, 2004), the definition of Catano et al. (2019) is retained to emphasize the behavioral dimension of management skills. These authors define competence as “a measurable attributes or behaviors that distinguish outstanding performers from others in a defined job context” (Catano et al., 2019, p. 155). Thus, the combination of all these elements forms the basis of a manager’s behavior and future performance (Bennis and Nanus, 1985; Parks, 1985; Kirkpatrick and Locke, 1991). For the sake of clarity regarding terminology, the term management behaviors will be used throughout this study, respecting the terminology used by each of the cited authors (e.g., management skills, management practices).

Thus, the model that comes closest to the concept of management behaviors is the competent manager of Boyatzis (1982), which presents a frame of reference making it possible to direct the actions of the manager. In addition to determining the management competencies necessary for performance (e.g., proactivity, logical thinking, self-control), this model also integrates the organizational context in which the manager operates (Boyatzis, 1982). Thus, this model evokes that through actions such as coordination, planning or decision-making, the manager participates in the realization and achievement of the objectives of the organization (Boyatzis, 1982). Although the Boyatzis model (1982) dates back more than 40 years and the labour market has evolved since then, it remains a precise model for guiding the actions of managers. In addition, it offers a diversity of behaviors that can be expressed by managers (Boyatzis, 1982). However, the next models presented will make it possible to better define management behaviors as concrete actions taken by managers in organizational settings.

Whetten and Cameron’s (2005) model, which is more recent, presents skills rather than competencies to conceptualize the construct of management behaviors. For these authors, management skills are characterized by behaviors that can be controlled, developed, or even interrelated (Whetten and Cameron, 2005). The results obtained in the context of their study present 13 management skills (e.g., leading positive change, motivating others, managing conflict) which are separated into four levels: personal; interpersonal; group; and communication skills (Whetten and Cameron, 2005). Since this model considers behaviors rather than personal characteristics, it is, therefore, closer to the daily activities and behaviors of managers, which underlies the relevance of the presence of this model in the conceptualization of management behaviors (Whetten and Cameron, 2005). However, this does not take into account the various changes that organizations may encounter, and which require, most of the time, a constant review of management skills to adapt to the context (Viitala, 2005).

In response to this need, Viitala (2005) integrates the notion of changes that organizations face in their daily operations. Her work supports the idea that managers must adapt their behavior according to the changes that operate in their environment, which may be of a financial, technological or any other nature. In the study of 794 Finnish managers, the author presents a hierarchy of six categories of competencies that managers can develop: (1) intrapersonal, e.g., character traits, self-image, tolerance for uncertainty; (2) interpersonal, e.g., adaptability of the manager in his relationships with others, ability to understand people and their behaviors; (3) leadership and supervision, e.g., ability to lead, support and engage people; (4) management, e.g., ability to achieve defined goals, to process information; (5) business, e.g., ability to provide vision, planning, use of resources; and (6) technical, e.g., use of tools, procedures, domain-specific knowledge. Illustrating her model in the shape of a pyramid, Viitala (2005) presents at the bottom of it intrapersonal and interpersonal skills, i.e., those which are more related to the characteristics specific to the individual and to his personal development and which require more effort to be developed. So, at the top of the pyramid are business and technical skills, which can be acquired more easily through education or work experience. For this author, the nature of management competencies refers both to all the experiences and knowledge acquired, to the skills, characteristics, social roles, values and even attitudes acquired by the manager throughout his career or his life. Viitala’s model (2005) is therefore one of the only ones to illustrate the fact that the manager’s competencies can evolve according to the context in which he finds himself. Moreover, notwithstanding the consideration of the characteristics of the context in the identification and development of the skills of managers, it turns out that many of these competencies are relatively generalizable and stable to all managers and organizations (Viitala, 2005).

The three models presented above reflect in themselves the conceptualization of management behaviors. Although this construct has been widely taken up and defined by various authors (e.g., Luthans, 1992; McGill et al., 1992; Flynn et al., 1995; Bloom et al., 2019), the models discussed in this study constitute reliable models still used in the literature (St-Hilaire, 2012; Lamsa and Savela, 2014). Within the present study, it is therefore relevant to retain the model of Viitala (2005) since it is the only one to consider that the competencies of managers can evolve according to the context or the changes that managers face in their daily tasks. Moreover, the hierarchical aspect of this model can be represented as a continuum going from more person-related skills to more task-oriented skills (Rifkin et al., 1999). In this sense, the closer the competency is to the base of the pyramid, the more fundamental it would be for the potential performance of a manager (Bennis and Nanus, 1985; Parks, 1985; Kirkpatrick and Locke, 1991). Thus, the hierarchy of competencies presented in the model of Viitala (2005) is particularly interesting considering that the competencies located at the base of the pyramid could appear more essential in a crisis context. Indeed, as described in the study by Panos et al. (2009) which looks at the qualities and characteristics of managers in the health sector in the context of a health crisis, in such a context, the actions of the manager must involve sensitivity and their decisions must consider the human factor. The use of Viitala’s model (2005) therefore appears particularly useful to answer the following research question: what management behaviors were put forward by middle managers in the health sector during the first wave of the COVID-19 crisis? Indeed, this model makes it possible to situate the behaviors expressed by managers according to the hierarchy of the six established competencies (Viitala, 2005) and therefore to determine whether the management behaviors expressed in a crisis context correspond to the categories of management competencies proposed by the model. Thus, the general objective of this study is to document the management behaviors used by middle managers during the pandemic, to better prepare and train managers for future crises. To do this, a qualitative approach was chosen to accurately represent the perspective of managers in this context.

## Materials and methods

### Design

The focus group method is an effective way to promote discussions between participants and quickly obtain qualitative information on a specific phenomenon (Stewart and Shamdasani, 2015). In this study, a pragmatic approach was adopted to meet the practical needs of managers in a crisis context, while also adopting an exploratory approach given the specific context surrounding the study. Given the impossibility of conducting focus groups in person due to the pandemic, the focus groups were conducted online in a synchronous manner. In this perspective, several authors have looked at the comparison between synchronous, asynchronous and face-to-face focus groups (Van Eeden-Moorefield et al., 2008; Murgado-Armenteros et al., 2012). The results of their studies confirm the same data quantity and quality obtained by a synchronous focus group compared to a face-to-face focus group (Murgado-Armenteros et al., 2012; Abrams et al., 2014; Boydell et al., 2014). In addition, important advantages of conducting online focus groups are to facilitate recruitment (Boydell et al., 2014; Cortini et al., 2019) and the self-disclosure of participants (Moon, 2000; Woodyatt et al., 2015). Therefore, considering the specific context of this study, it was relevant to conduct an online focus group since not every manager could have been in person due to the organizational constraints in place in the health sector. Thus, the use of focus groups made it possible to offer a safe space to managers to encourage discussions on the challenges encountered and the expression of their concerns in the context of the pandemic. Even if, in the literature, the ideal number of participants is not unanimous (e.g., Cortini et al., 2019), several focus group technique scholars suggest 8 to 12 as an ideal number (Stagi, 2000). It is recognized to choose the number of participants in focus groups according to the theme and the context of the research (Cortini et al., 2019).

### Participants and procedure

The recruitment of participants for this study is the result of an initiative of the human resources department of a university hospital center in the province of Quebec, Canada. To do this, an e-mail invitation was sent to all the hospital center’s middle managers to participate in weekly discussion meetings. The purpose of these eight meetings was to promote the sharing of strategies for managing and adapting to the context of the pandemic, to learn about leadership skills and to provide a space for support between peers. Thus, to be eligible for the study, participants had to be middle managers working within the hospital center at the start of the pandemic. To maximize the heterogeneity of the professional realities of the healthcare environment during the first wave of the pandemic and to obtain a broader and more complete understanding of the different behaviors expressed by managers, the participants who took part in the study came from different hospital sectors, such as management of nursing, technical services, human resources, hospital services, etc. A total of 37 middle managers took part in the study, including 11 men and 26 women. On average, there were 10 participants present at each meeting, which is in line with the literature on the use of focus groups which defines that it takes around 6 to 12 people to generate a dynamic in the interactions, shared meanings as well as points of divergence between members (Simard, 1989). Participants ranged in age from 31 to 55 (*M* = 43.41; SD = 6.95) and had an average of 6 to 10 years of experience in a management position. Finally, all the participants were present on average 2.19 times over the eight meetings, including 21 of them who participated in only one meeting.

The eight focus groups took place in French, virtually via the Zoom platform, each week, for eight consecutive weeks starting from April 2020 until June 2020, i.e., during the first wave of the pandemic in Quebec. Each focus group lasted about an hour and was recorded and then transcribed verbatim. During each meeting, the same two facilitators were present. One was asking the participants questions while taking notes, while the other managed participation and introduced and concluded the meetings. Using an interview guide, various questions related to the research objectives were asked to the participants to guide their discussions around the theme of leadership and management behaviors during the pandemic. For example, the following questions illustrate the theme covered by this study: “What have you changed in your managerial approach? What do you think is the most important thing to do as a manager? What have you learned so far about your leadership style? “Also, throughout the focus groups, the facilitators used probing questions to clarify more ambiguous answers. For example, if a participant gave a vague answer to a question or answered with a general statement (e.g., “we prepare the teams in this way), the facilitator asked him to give specific examples of behaviors actually expressed to support this affirmation (e.g., “what is it in practice…how do you go about preparing your teams in relation to what you are saying?”). Researchers were then able to better analyze and understand the behaviors expressed by managers in the context of a crisis.

### Ethical considerations

It should be noted that the present study is part of a larger joint research that has already obtained the approval of the healthcare facility’s research ethics committee. Indeed, the first collection of quantitative data on the adoption of leadership behaviors among managers was carried out in the fall of 2019 and a consent form was signed by all participants. When the Covid-19 crisis started, seeing the opportunity to contextualize the experience of managers during this period, a second qualitative data collection was carried out in 2020. This second data collection has also been approved by the ethics committee. To protect the confidentiality of the participants, it was mentioned in the invitation sent to the managers that the virtual meetings would be recorded, that they would not be broadcast, that the exchanges would remain confidential, and that no information would identify the people who took part in the study. In addition, at the start of each meeting, the facilitators mentioned again that the meetings would be recorded and that the content would be analyzed by the researchers to follow up on the initial research in which several of the participants had participated in the fall of 2019, thus it is, therefore, an implicit consent to participate in the study.

### Analysis

Thematic analyzes were carried out within the framework of this study using the MaxQDA 2022 software. This method of analysis makes it possible to proceed systematically to the identification, grouping, and precise examination of the themes addressed in each context (Mucchielli and Paillé, 2008). Moreover, this type of analysis also makes it possible to draw reliable, valid, and generalizable conclusions (Lincoln and Guba, 1985; Elo and Kyngäs, 2008; Elo et al., 2014).

To do this, the thematic analysis method described by Braun and Clarke (2006) including six steps, according to a recursive and continuous process, was retained. To carry out the first step, which aims at becoming familiar with the data to prepare the data for the analyses, the recordings of the focus groups were first transcribed in verbatim form while checking for possible transcription errors. Then, the transcripts were read several times to obtain an overall impression of the data collected and to ensure that the transcripts accurately reflected the discussions held by the participants before starting the coding stage (Halcomb and Davidson, 2006). The transcripts were then exported into the MaxQDA 2022 analysis software. In the second step, from the theoretical model retained in the present study, i.e., the hierarchical model of management skills by Viitala (2005), codes were generated in a deductive way, i.e., based on the literature, to start the coding stage. The researcher first carried out calibration exercises with her colleagues to clarify the definitions of the codes created to facilitate coding. Each sentence, paragraph or passage that represented an idea named by a participant was considered a unit of meaning. The smallest unit of meaning considered was a sentence containing at least one verb and one subject. In addition, the segments of the verbatims that were not related to the management competencies were excluded from the coding process to keep only the segments related to the defined codes and to ensure better content validity. Then, the coding of the data was subjected to a validation process of inter-rater agreements to ensure that the units of meaning coded represented the data. Inter-rater validation is defined by the consistency in which different analysts attribute according to the same coding scheme, the same code to a randomly given segment (Mukamurera et al., 2006). One of the co-authors, trained in qualitative analysis, coded a randomly selected focus group. This first part of the inter-judge validation process made it possible to raise some ambiguities in the definitions of the codes and made it possible to specify certain elements, in particular related to the division of the coded segments, i.e., the defined unit of meaning. To reduce the coding variability, the percentage of code overlap on the segments selected by the coders was set at 50% to allow some variation in the division of the coded segments. This way of proceeding seems consistent with the statements made by Lombard et al. (2002) who explain that there are several ways to calculate the inter-rater agreement. Following the discussions held with the coder having the role of judge on the elements named above, a second discussion group was codified. A Kappa of 0.69 was calculated by the MaxQDA software (Kappa from Brennan and Prediger, 1981) and a percentage agreement of 73.45% was obtained. This coefficient indicates a “substantial” strength of agreement, according to the scale of Landis and Koch (1977). These results support the validity of the study.

Subsequently, to identify the management behaviors to answer the research question, the coded segments corresponding to the management competencies of Viitala’s model (2005) were extracted from the analysis software in order to group and classify them inductively into categories of management behaviors, which corresponds to the third stage of Braun and Clarke (2006). The inductive approach allows the creation of codes, categories or themes derived from the data (Blais and Martineau, 2006). Then, in the fourth step of the thematic analyses, for each grouping of segments, several statements of behavior categories were written and modified, making sure to clearly understand their meaning and to respect the following criteria: reflect all the content of the grouping; elaborated by simple, clear, and precise statements; formulated positively; elaborated in a complementary manner with respect to the other statements; and worded accurately without distorting the words. Discussion sessions were held with colleagues from the research team to ensure a common understanding of the results and their credibility. The concept of credibility refers to the accuracy of the results and the concordance between the empirical observations and their interpretation (Paillé, 1994). According to Miles and Huberman (2003), this type of discussion is an effective way to reach an “intersubjective consensus,” i.e., an agreement between the different researchers who took part in the analyses. In the fifth step, proofreading and verification of all the categories of management behaviors defined were carried out to ensure the consistency and accuracy of the analyses. Finally, the sixth and last step of Braun and Clarke (2006) consisted in writing the final version of the management behaviors and providing the associated excerpts to demonstrate the prevalence of the categories of management behaviors defined.

Several aspects make it possible to establish the validity of this study, such as: considering the biases held by the researchers when collecting and interpreting the data; verification of the accuracy of the results obtained; and maintaining discussions with all researchers to improve the accuracy of data analysis. In terms of reliability, the researchers met regularly to discuss the results obtained and in the event of uncertainty during the analysis of the results, the principal researcher consulted her colleagues to obtain different opinions.

## Results

This section describes and interprets the results obtained in response to the following research question: what management behaviors were put forward by middle managers in the health sector during the first wave of the pandemic? More specifically, the study aims at describing the experience of 37 middle managers in the Quebec health sector and at identifying the management behaviors used by them during the first wave of the pandemic. To do this, the hierarchical model of management competencies by Viitala (2005), which presents six categories of managerial competencies considered important for the role of a manager, was retained. A total of 21 management behaviors have been identified and grouped according to these six managerial competencies.

First, an overview of the results is presented (see Table 1). For each competency in the Viitala (2005) model, the associated management behaviors are stated, defined, and accompanied by excerpts from the related focus groups. Each extract is associated with the number of the focus group in reference (e.g., FG2) and the number of the participant interviewed is attached to it (e.g., P3). Moreover, the presentation of management behaviors does not consider the frequency of the stated behaviors, i.e., the number of times the behavior was reported by the participants, given that the study is interested in documenting all the behaviors management put forward during the first wave of the pandemic. In addition, as soon as a management behavior was named and had been used by one of the managers during the crisis, it was retained. The results, therefore, highlight the overall portrait of all the management behaviors that were used by middle managers during the first wave of the pandemic.

**Table 1:** Management behaviors issued in a COVID-19 crisis situation related to Viitala’s management competencies model (2005).

Management competencies	Management behaviors
1. Intrapersonal	1.1 Setting its limits
	1.2 Adjust their way of interacting with others
2. Social	2.1 Listening to the teams
	2.2 Distribute the hours worked in the team
	2.3 Discuss more personal matters
3. Leadership and supervisory	3.1 Be present and available to the teams
	3.2 Consult teams at the right level to achieve common goals
	3.3 Manage the work overload and rhythm of the teams
	3.4 Explain the meaning of decisions
	3.5 Set an example for the teams
	3.6 Disseminate information transparently to teams
4. Management	4.1 Imposing directives on the teams
	4.2 Manage conflicting information
	4.3 Manage the gap between directives and the reality on the ground
	4.4 Standardize work methods
5. Business	5.1 Develop long-term plans
	5.2 Analyse the work methods of other sectors to improve their ways of doing things
	5.3 Reorganise the work according to the priorities defined
	5.4 Obtain the necessary resources to meet needs
6. Technical	6.1 Use their knowledge of project management
	6.2 Use their knowledge of civil security to better convey information

### Intrapersonal competencies

This competency refers to the motivations, values, and self-image of the manager (Viitala, 2005). In the context of the study, this competency translates into the manager’s ability to explain the choice of his actions through elements related to his natural tendencies. Through the focus groups, two management behaviors relating to this skill have been documented.

#### Setting its limits

During the focus groups, the managers mentioned the importance of setting their limits to preserve their life balance despite the particular context in which they found themselves. Indeed, although the situation meant that they were overwhelmed at work, the managers had to set their limits, particularly in terms of their working hours, as described by participant P3, to be able to save energy to get through the crisis.

I tell myself what I also have to work on, is to be able to set my limits because no one will survive. Then, one thing to adapt following our exchanges is really that, to be able to set limits and then say: “Well no, I can’t, it’s too much”. I don’t have enough minutes in my day, I have to postpone it. (FG4, P3)

#### Adjust their way of interacting with others

According to the P2 and P4 participants, they had to adjust the way they interacted with others to adapt to everyone's pace. Indeed, they realized that in a crisis context, some people react differently than usual, for different reasons (e.g., stress, exhaustion, anxiety), which means that self-management becomes an important element in facilitating relationships with others.

In my style of leadership, I’m definitely a very colorful person, who takes up space and now, I just learn to measure myself then to (breathing) because I realize that even if I am filled with goodwill, even though I want to pay attention to everyone, well I just need to take it ‘smoother’. (FG1, P2)

I have to talk to myself; I have to manage myself because I feel that the team is tired too. (FG4, P4)

### Interpersonal competencies

Interpersonal competence is defined by the manager’s ability to maintain relationships with others (Viitala, 2005). This competency also refers to the manager’s ability to understand the behavior of others, to put themselves in their shoes and communicate with them. In this study, middle managers put forward three management behaviors related specifically to this competency.

#### Listening to the teams

The participants shared during the focus groups the need to listen to their teams to see how they were doing in the particular context in which they found themselves. The participants mentioned that they had to be attentive to the difficulties or personal issues that some of their team members might be experiencing to prevent the more personal aspects of certain members from spreading to the whole team.

Yes, to have the necessary listening. Me, a bit like [name of participant 4], I took my airtime that I had developed with my teams to do “top-down” but to go more into “How are you?”, then to chat with them others then yes, to go in what is more individual well to pack it down to bring it with this person alone so that it is not redistributed in the whole group either. (FG3, P2)

#### Distribute the hours worked in the team

In addition to listening, the managers also mentioned that they were very concerned about the well-being of their teams, more specifically, to ensure that their teams maintained a work-life balance despite the crisis context. From the words of participant P8, we understand that he questions his employees on the number of consecutive days spent at work to see if he cannot give certain holidays to those who have worked too much overtime to allow them to rest. This behavior illustrates that even in a crisis, the manager still ensures the physical and psychological well-being of his employees beyond the work overload that accompanies them in this particular context.

So, you know, I paid attention to people there a lot in terms of “quality of life”. You know, people I’d been seeing for a while: “Okay, how long have you been here? What are you doing from now on? How many days in a row have you been working? You know, at one point we were asking”. Well, ok. Should I bring in such and such an extra person? It would be his 8th day. No, we are thinking of another option. You know, we won’t burn everyone. We try to manage that basically as best as possible to avoid spreading the workload amongst all the group. (FG2, P8)

#### Discuss more personal matters

During the first wave of the pandemic, in the health sector, the focus was mainly on getting the job done to get through the crisis. To relax the atmosphere of the teams in such a context, the managers had to find ways to change the mindset of their teams. In fact, as mentioned by the participants, talking about aspects related to their personal life allowed the teams to see how everyone experienced the first wave of the pandemic outside the workplace, which did well. We then understand that interacting with their teams on elements other than the work itself is a way for managers to support their teams to get through this more difficult period.

We try to talk and then, in fact, I spend time chatting with people. I tell them a bit about how I experience it at home, then people explain to us how they experience it at home, and it feels good just to talk about it. It’s not necessarily to talk about work but to talk about everyday life a little and then to see a little about the different situations… sometimes crazy but I think talking about this everyday life is good for people. (FG3, P7)

### Leadership and supervisory competencies

Leadership and supervisory competence refer to the manager’s ability to orient, influence and direct his teams while involving them in the pursuit of defined objectives (Viitala, 2005). This competency also refers to the means that the manager takes to lead his teams to achieve the goals set. This research has identified six management behaviors related to this competency.

#### Be present and available for the teams

As mentioned by participant P1, managers had to be more present with their teams to facilitate change management and to ensure better control given that in a crisis context, there is often new information and directives to follow. By being close to their teams, it was then easier for managers to intervene quickly and answer their questions so that they could continue their work.

Everything stopped then we were 100% with our employees then it really made it easier for change management because we had the pulse from hour to hour then we were able to intervene when we felt we were losing control. (FG4, P1)

#### Consult teams at the right level to achieve common goals

During the crisis, managers reported that they tried to consult with their teams to try to respond as best as possible to their different needs and concerns. However, the managers found that they had to put a certain limit considering that they could not satisfy everyone’s needs. They had to find a happy medium to meet the main needs of their teams without adding too much information or additional tasks at the risk of further complicating the achievement of the defined objectives.

You know, it’s hard to know, as a manager, how far we’re going in this because in fact, if we listen to everyone, at some point, there will be so many additional tasks that people will be lost, they won’t know what alignment is, they won’t really know where they are going. […] The question I asked them was: “In your opinion”, do you think we are doing too much or not enough? and then ask them: “What do you” … what are the elements that you find that we do not do enough and then what are the elements that you find that we do too much? […]. So, we have to try to temper between the 2 and that, it’s not easy to try to do enough but not too much and then also to be able to keep our teams afloat. (FG6, P4)

#### Manage the work overload and the rhythm of the teams

According to the participants, they recognized the overload of work that was imposed on their teams during the crisis, in particular, because of the large quantities of information and the new directives that they were given. The teams, therefore, had little time to assimilate this set of information. As mentioned by participant P3, he explained that regarding this overload, he tried to respect the rhythm of his teams and give them some time to integrate new information before giving them something new.

Well, I think we have to allow them to acquire everything we have pushed in the last few months. It’s already very exhausting and you know, we haven’t been able to do any change management in the last few months. We had an order: “Well let’s go gang, we put the trajectories. Now, we put up walls, we put up doors, we separate”. So there, you have to give them time to learn how to navigate in there before arriving with a new “top-down”. (FG4, P3)

#### Explain the meaning of decisions

During the first wave of the pandemic, the usual functioning of the teams was modified, due to the addition of new tasks related to Covid-19. From the comments of the participants, we understand that some teams had difficulty understanding and following the decisions made by management. They were looking to their managers for explanations of these new rules, which changed often. As mentioned by participant P1, managers then had to explain to their teams the reasons behind these decisions to make them understand the meaning or the objective of this new operation.

We really have to try to explain why, why we made this decision. So, I’m very much in that dynamic. Sometimes it’s not easy. We have to re-explain a lot and then we sometimes have to take people individually to really make them understand the why of a decision, the why also that time is important… at the moment we are at it, we have to make that decision like that. (FG1, P1)

#### Set an example for the teams

According to the participants, it is also important for a manager to set a good example for his teams in a crisis context. More specifically, by setting good examples to follow, the manager encouraged his teams to adopt the behaviors he wanted to see by demonstrating them in his own attitude. As mentioned by the P2 participant, he showed his team that it is possible to resume a “more normal” rhythm of life to show the importance of preserving a good balance of life despite the particular context.

It’s to show them that I’ve always… I’ve always told them that it's important that they have a balance, that they have to do it, right there, by setting an example with them to others well yes me too now I have normal working days then I will be there with you guys today 9 am to 5 pm then Thursday I will be there 7 am to 3 pm but I will be there for 8 hours, not 12 hours. (FG3, P1)

#### Disseminate information transparently to the teams

As mentioned by the P2 participant, he reported that being transparent with his teams, or communicating openly and honestly with them, was a means used to facilitate the management of information in a crisis context. Indeed, by remaining true to the information that the manager had, he did not let his teams claim that he was hiding information from them, but rather that he was experiencing the same situation as them.

So, I think it’s to be transparent […] I think that transparency, at least for me, is what is the best for me now… to be authentic in all of this. Then when we don't have the answer, we simply don’t have it. (FG3, P2)

### Management competencies

Management competence is defined by a manager’s ability to achieve set objectives (Viitala, 2005). Furthermore, this competency requires the manager to process the information present in his environment to guide the teams towards the achievement of results and to implement the necessary measures to achieve them. As part of the study, four management behaviors were identified.

#### Imposing directives on the teams

As reported by participant P5, in a crisis context, managers came with the directives they received from top management and imposed them on their teams without seeking their opinion for lack of time. This so-called “top-down” management approach allowed the manager to apply directives more quickly in a context where time was precious for them.

We are used to also taking the pulse on the ground, asking the field, in the things we do: “Does that make sense? How do you see that?” And then, it was no longer that at all. We came up with the way to do it and then that was how we did it. So, it was a shock for my management style, but also for my employees who, basically, they weren’t used to it […]. In the “top-down”, it’s that we don’t have time to go through that process and then they are told, “That’s how it is, that's how it is”. (FG1, P5)

#### Manage conflicting information

In their day-to-day management, managers received a lot of information that changed quickly and was sometimes contradictory to the rest of the information received. Therefore, they had to continually adjust and filter all the information to retain the most important ones and forward them to their teams.

I did, the scrums at the end of the day, on a voluntary basis, because during the day, the information changed so much and, one moment, I gave them information and the next day, it was no longer relevant. (FG1, P7)

#### Manage the gap between the directives and the reality on the ground

In addition to having to continually adjust to new information received, managers also stressed that they had to bridge the gap between the directives received from top management and the way in which they were to apply these directives in the field. Indeed, managers had to take into account the reality of their environment so that the directives made sense once in the field. The P4 participant also mentions that he often found gray areas, and ambiguities in the way of applying the directives, which added a level of complexity to make the directives concretely applicable. The challenge for the managers was therefore to succeed in figuring out how to easily apply the directives and avoid any confusion in the field.

One of the roles we have is to apply it with the team and then how, concretely, we are going to apply it and now, there is a whole gray area that settles between what is asked then the how are we going to apply it with the team because now, there are lots of little details that people will think about then that you have to look at how we’re going to apply that. (FG6, P4)

#### Standardize work methods

Through the focus groups, the managers also mentioned that they meet regularly to find ways to standardize the application of the directives. As mentioned by participant P13, by standardizing their ways of doing things among managers, they made sure to have a common understanding of the directives and to convey them in the same way to their teams to avoid creating even more confusion than there already was.

So, the application of these directives that we received well it was always necessary to make sure that they were more uniform. Then yes, people compare themselves, so we tried to have management meetings to try to bring together that, discussing the directives and then applying them all in the same way, but that hasn’t always been easy of course. (FG7, P8)

Even at the beginning, we met every day, every shift to make sure we understood the instructions and then that we got them down the right way. (FG7, P13)

### Business competencies

This competency refers to the manager’s ability to plan, develop strategies, and establish an overall vision and direction (Viitala, 2005). This competency allows the manager to have an overview of what needs to be implemented to follow the established direction. In the context of this study, four management behaviors relate to business competence.

#### Develop long-term plans

According to the managers, long-term planning is an important aspect in a crisis context. Indeed, as mentioned by participant P7, the development of plans allowed managers to project themselves in time to set their objectives and organize themselves accordingly. Planning in a crisis context also allowed managers to reduce the ambiguity that could be associated with a situation in a crisis context.

Then I have the impression that redoing a plan… an organizational plan for the next few weeks, next months, is going to be really, really important so that the unknown becomes more of the known. (FG3, P7)

#### Analyze the work methods of other sectors to improve their ways of doing things

Another management behavior that emerged during the focus groups is to explore the working methods used in other sectors to better understand how they work and see if it would be possible to adapt their sector based on the observations noted. This form of mutual assistance between colleagues allowed them to draw conclusions from what was done by other managers to improve work processes in a crisis context.

Then with the links, we made, well, I watched with people in the clinical labs how they had worked to settle in… with the benches and then all that. So we … with the other exchanges we had previously, it allowed us to connect with those who … well you know, the clinical labs that … [name of participant 4] it continued during COVID, it was on a roll “full pin” so he was in it then he had had the experience so we were able to have a bit of this … how I would say, “heads-up” so as not to start from scratch and then adapt to that. (FG6, P3)

#### Reorganize the work according to the priorities defined

Faced with the first wave of the pandemic, managers found themselves with an overwhelming number of tasks to carry out, and this, while being pressed for time. As reported by the P4 participant, priority management has become a central point in the way they organize their work to keep their energy to deal with more urgent files.

The lack of time then me too, that’s it, it’s the management of priorities as [name of participant 3] has just said. It is that we must review our priorities. We have to review… I have to review my priorities because I… we won’t be able to do everything that is asked of us so it’s going to be to see what is most urgent and then which is the highest priority. (FG4, P4)

#### Obtain the necessary resources to meet needs

As mentioned by participant P8, managers had to increase their resources in order to be able to deal with the crisis since otherwise, they would not have been able to meet the demands. The crisis context has therefore highlighted the urgency of acquiring additional resources (e.g., human resources, material resources) to meet the needs raised by the pandemic in the health sector.

But, basically, we here at the laboratory level, the necessary resources there, we had to add a lot to be able to produce. You know, at the level of medical biology, the laboratory sectors there, everyone responded well to the call. We really had help from several laboratories in other hospitals there, you know. But now, these people are currently producing at full speed. They are really well integrated into the team. So, we had to increase the staff to be able to meet the demand yes. (FG4, P8)

### Technical competencies

Technical competence refers to a manager’s ability to use instruments, procedures, or tools in a specific area (Viitala, 2005). This competency usually represents the use of knowledge from the manager’s area of specialization (e.g., finance, engineering). In the context of this study, two management behaviors related to this competency were identified.

#### Use their knowledge of project management

Managers who have taken project management training in the past have reported that the knowledge acquired through this training has been very useful to them in structuring and organizing themselves in their daily operations to deal with the crisis. This type of knowledge could therefore be used by all managers to facilitate their management in a crisis context.

We have management training in relation to project management, so we already had our minds “set” on this principle, but it could… I don't know if you offer it in your training booklet, training for managers, even for newcomers, project management […] in the sense that it’s very useful for everyone, and at the same time it gives us a way of maybe facing our challenges by structuring ourselves. (FG6, P11)

#### Use their knowledge of civil security to better convey information

According to the participants, the civil protection model was useful in structuring the descent of information from top to bottom. Indeed, this model allowed managers to give a line of conduct to teams to avoid confusion in the field. However, according to the comments of participant P8, the civil protection model deserves to be further acquired among all managers to ensure a common understanding of it.

I ended up using it in my management in terms of leadership to say: “Here, there really is a structure, it’s emergency preparedness that must make those decisions, in those committees, we must…you know, to try to really get these…you know, to really explain on the ground how it works because even if…still you know, if I was a bit lost or confused in there, fine people on the ground were even more…of why such a decision is made and everything… (FG1, P1)

Then the civil security structure, I think that is not necessarily acquired for everyone. […] Within our imaging branch, it was really the civil security structure that was not well understood, in the sense of how the encounter chain is going. (FG7, P8)

Thus, the results obtained made it possible to answer the following research question: what management behaviors were put forward by middle managers at the start of the pandemic? Furthermore, through the management competencies model of Viitala (2005), it is possible to understand that according to the six categories of competencies defined by the author, certain categories have been used more frequently or put forward during the COVID-19 crisis.

## Discussion

The objective of this study was to generate knowledge by documenting the different management behaviors expressed by middle managers in the Quebec health sector at the start of the pandemic. Following a qualitative approach, eight focus groups were carried out virtually and made it possible to identify different specific management behaviors, through all the testimonies made by the managers who participated in the study. By choosing a qualitative approach, this study makes it possible to highlight the experience of middle managers in this very particular context of the COVID-19 crisis.

The results show that all the competencies proposed in Viitala’s model were used by middle managers in the Quebec health network during the COVID-19 crisis, thus supporting the relevance of this model. This study thus made it possible to transfer the six competencies of Viitala (2005) into 21 concrete behaviors applicable to managers. The results of the present study are in line with those obtained in the study by Caringal et al. (2021) which examines the perspective of employees during the pandemic. Indeed, these authors highlight the following behaviors as having been particularly useful: supportive behaviors, behaviors focused on decision-making, transparency, and openness in communications, and behaviors focused on collaboration and consultation. Thus, the results of the present study echo the behaviors identified in the study by Caringal et al. (2021), which supports the existing literature. On the other hand, one of the behaviors that emerged in the study by Van Wart and Kapucu (2011), but which was not observed in the present study, is delegation. Indeed, the managers who took part in this study did not promote this management behavior. This can be explained by the fact that in the health sector, during the pandemic, the teams were exhausted, overworked and understaffed in several departments. Thus, in such a context, it appears more difficult for the manager to delegate certain tasks.

In addition, the results show that in a crisis context, the management behaviors that have been most reported are more team-oriented (e.g., being present and available for teams) rather than task-oriented (e.g., using knowledge of project management). In fact, the results indicate that it is the management behaviors related to leadership and supervisory competencies that have been mostly put forward by middle managers and that, conversely, the management behaviors related to technical competence were less noted. According to James and Wooten (2010) and Thach (2012), leadership competencies are essential in crisis management to implement the decisions made while maintaining the trust of stakeholders. Moreover, during turbulent times, such as a pandemic, the role of the leader becomes more important as employees feel more insecure, dependent and stressed. They then seek more social cues rather than situational ones and are therefore more likely to turn to their leader (Shamir et al., 1993; Kets de Vries, 1998; Shamir and Howell, 1999). In short, according to the hierarchy of management competencies presented by Viitala (2005), it would seem that the skills located at the base of the pyramid, in particular interpersonal and leadership competencies, are fundamental competencies in a crisis context, which goes in the same meaning as other studies on the subject (e.g., Panos et al. 2009).

### Theoretical and practical contributions

First, this study shows the relevance of still using Viitala’s (2005) model in the context of a crisis, such as that of COVID-19. Indeed, this model is relevant since the behaviors identified refer to all six competencies of the model. As mentioned by Lamsa and Savela (2014), this model is particularly interesting because it covers a wide spectrum of competencies, whether related to “hard” or “soft” skills. Thus, a great diversity of behaviors can be listed thanks to this model.

Second, the results of the study make it possible to deepen the model of Viitala (2005) by making the model more specific through concrete behaviors. Indeed, the study made it possible to put forward 21 management behaviors specifically used by middle managers during the COVID-19 crisis (e.g., imposing directives on teams, explaining the meaning of decisions, his limits). This study supports the current literature that advocates the use of specific management behaviors in relation to leadership, which appears as a broader concept (St-Hilaire, 2012; Gilbert et al., 2017).

Third, the results of this study contribute to expanding the literature on the context of the COVID-19 pandemic. Considering that studies on COVID-19 have been carried out in different countries (e.g., Bajaba et al., 2021; Caringal et al., 2021), the Quebec context also seems relevant to investigate to better understand how this health crisis was managed by managers around the world. Indeed, it is important to note that crisis management may differ from one cultural context to another, where different factors must be considered to understand an organization’s response to the threat of a crisis (Elsubbaugh et al., 2004).

Fourth, at the methodological level, carrying out this study made it possible to offer middle managers a safe space to discuss with colleagues the difficulties encountered in the context of the pandemic. Furthermore, the use of focus groups made it possible to break the isolation by offering peer support and identifying management and adaptation strategies in such a context. Thus, the present study contributed to taking care of the psychological health of managers so that they, in turn, are willing to take care of their teams. As described in the study by St-Hilaire and Gilbert (2019), the mental health of managers must become an organizational priority considering that it can affect the ability of managers to adopt good management practices for their teams.

Finally, the results of this study guide practitioners on the importance of competencies development for managers, especially in the context of crisis. Indeed, the results underline the relevance of the set of competencies proposed by Viitala (2005) and support the fundamental aspect of certain competencies such as interpersonal and leadership competencies. The results of the study also make it possible to guide trainers and managers toward the adoption of specific behaviors to promote the integration of these.

### Limits and avenues for future research

Although this study presents scientific and practical contributions, it is important to consider certain limitations that support reflections on avenues for future research. First, the results of the study make it possible to document the management behaviors put forward by managers during the Covid-19 crisis, but do not make it possible to establish whether these behaviors had real positive impacts. To compensate for this lack, it could, in particular, be relevant to conduct interviews with employees in order to identify the management behaviors found in this study, which according to them, were the most helpful in dealing with the crisis or to carry out a study with a quantitative design in order to deepen these management behaviors by means of indicators (e.g., number of patients seen, reduction in waiting time, number of files processed). Secondly, it is obvious that certain limits must be mentioned surrounding the process of carrying out and interpreting the interviews conducted with information relating to a phenomenon while allowing the participating facilitators. Indeed, the use of focus groups makes it possible, among other things, to provide significant quantities to ensure control over the alignment of questions (White and Dotson, 2010). However, this method of data collection involves certain limitations such as the presence of bias among researchers. Also, since this research is part of a larger study, some participants had already answered a questionnaire for the first measurement time (8 months before) for the quantitative part of the study. However, other than this past experience, the researchers had no other ties to the participants. Furthermore, although the focus group method was chosen for this study, it would be possible to find different results with other types of qualitative methods, such as field observations. Third, since the focus groups were conducted with a single hospital center in the province of Quebec, with a small sample of 37 middle managers, it is possible that the management behaviors identified were different in other hospitals since certain regions of Quebec were more affected by Covid-19 during the first wave of the pandemic. Moreover, these establishments may not all have the same organizational culture. Indeed, several studies have looked at the relationship between organizational culture and crisis management. Among these studies, Bea (2011) found that organizations with fewer reported incidents foster a more crisis-sensitive culture through various activities, such as educating employees to detect crisis signals and assess risks or security practices to prevent crises. Lastly, this study focuses only on a three-month period at the beginning of the pandemic. Therefore, as the pandemic evolved, several changes took place in the management of the crisis. Not only in terms of management, but the virus itself also changed and different variants appeared as the months passed. Moreover, at the beginning of the pandemic, that is, in the 3 months when the study took place, vaccines had not been developed yet and wearing a mask was not mandatory like other sanitary measures that were introduced later. Thus, several elements have evolved since the beginning of the pandemic, and it would have been interesting to observe managers’ behaviours over a longer period and see for example the differences in the managers’ experiences before and after the vaccine use. As a matter of fact, some authors have reported the extent to which COVID-19 vaccination has favourable effects on individuals’ psychological well-being as others have reported that vaccine hesitancy and anxiety have become increasingly important during the COVID-19 pandemic (Nguyen, 2021; Bullock et al., 2022; Nazli et al., 2022). Therefore, it would have been also interesting to look at other aspects of the reality of managers beyond 3 months at the beginning of the pandemic.

Furthermore, the results of this study also highlight possible avenues for future research. Since medical sectors were differently affected by the COVID-19 pandemic, managers who participated in this study explained how their sector was affected by the virus at the beginning of the pandemic. Therefore, some mentioned that they were much busier due to the nature of their sector (e.g., emergency, intensive care) and on the other hand, some mentioned a slowdown in their service due to the transfer and adjustment to telework or simply because of the discontinuation of certain projects. Many studies do report that intensive care units were overwhelmed and challenged on multiple fronts during the pandemic (Goh et al., 2020; Zangrillo et al., 2020; Gualano et al., 2021). Thus, it could be interesting to look at the differences encountered by different medical sectors during the pandemic. Also, given that the present study is set in the context of a changing crisis over time (COVID-19), future research could focus on tracking the evolution of managers and their management behaviors through the pandemic or other crisis that evolves over time. In fact, Pearson and Mitroff (1993) developed a model that presents the evolution of a crisis according to five stages: (1) detection of signals (2) preparation and prevention (3) limitation of damage (4) recovery and (5) learning. Therefore, it could be interesting to group and identify the management behaviors put forward during the Covid-19 crisis according to these five stages to increase the precision of the results. Indeed, although the results of this study focus on the first wave of COVID-19, it seems possible to detect if those certain behaviors are representative of certain stages (e.g., planning to resume activities, reassuring teams in recovery). Moreover, a study by Wooten and James (2008) explored leadership competencies according to the stages of a crisis developed by Pearson and Mitroff (1993), in 20 companies that had experienced a crisis (p. accident, scandal, incident related to health and safety) between 2000 and 2006. With this in mind, it could therefore be interesting to check whether the management behaviors expressed during a crisis (e.g., the pandemic) are similar to the competencies identified within their study.

## Conclusion

This study has made it possible to understand and identify the management behaviors put forward by middle managers in the Quebec health sector during the first wave of COVID-19. More specifically, the results highlight specific management behaviors related to each of the skills identified by Viitala’s model. Therefore, the results support the relevance of using this model of management competencies in the context of a crisis, such as that of COVID-19. This study has therefore made it possible to highlight the importance of the role of middle managers since they act as creators of meaning and facilitators, particularly in a crisis context. Considering that other crises are likely to come in connection with global warming, it is important that managers are increasingly prepared to deal with this type of situation. Given this observation, it is even more relevant to prepare managers so that they acquire the necessary competencies and can adopt the right behaviors for the effective management of these possible crises.

## Data availability statement

Access to the data is restricted to protect confidential information. The data could be available upon request with permission of the healthcare facility and participants. Requests to access these datasets should be directed to M-HG, Marie-Helene.Gilbert@ulaval.ca.

## Ethics statement

The studies involving human participants were reviewed and approved by Comité d’éthique de la recherche de l’Université Laval and the study protocol was approved by the healthcare facility’s research ethics committee. The participants provided their written informed consent to participate in this study.

## Author contributions

MM conducted the analyses and took the lead in the writing and revision process. M-HG is the principal investigator of this project and conducted the data collection. P-SF and JG contributed to the reflection and orientation of the article. FB contributed to the analyses by conducting the inter-judge agreement. All authors contributed to the article and approved the submitted version.

## Funding

This study was funded by Fonds de recherche du Québec – Société et culture (FRQSC), Grant 2018-NP-206621.

## Conflict of interest

The authors declare that the research was conducted in the absence of any commercial or financial relationships that could be construed as a potential conflict of interest.

## Publisher’s note

All claims expressed in this article are solely those of the authors and do not necessarily represent those of their affiliated organizations, or those of the publisher, the editors and the reviewers. Any product that may be evaluated in this article, or claim that may be made by its manufacturer, is not guaranteed or endorsed by the publisher.
